# Supplementary material for: Myogenesis in C2C12 Cells Requires Phosphorylation of ATF6α by p38 MAPK
Source: Biomedicines. 2023 May 16;11(5):1457. doi: 10.3390/biomedicines11051457 (PMC10216283; doi:10.3390/biomedicines11051457)
Supplement: Supplementary file 1 [file biomedicines-11-01457-s001.zip › biomedicines-2308593-supplementary.pdf]

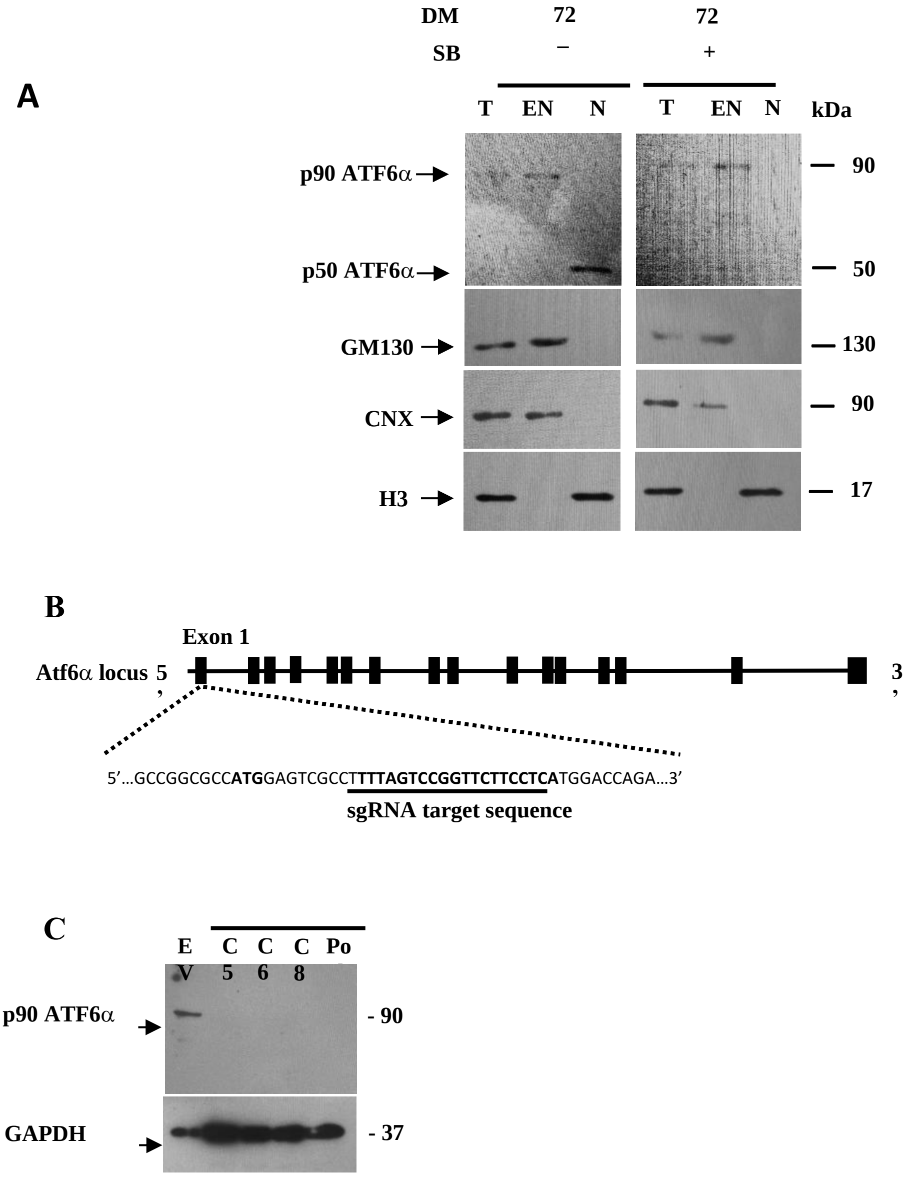

Figure. S1. Supplementary material for Figures 3 and 4. Supplementary material for Figure 3: (**A**) Western blots showing endogenous ATF6 protein localization in subcellular fractionation from C2C12 cells; Three distinct fractions corresponding to the extra nuclear fraction (EN) and the nucleus fraction (N), total fraction was indicated with T. The GM130 and CALNEXIN (CNX) were used as markers of the EN, and H3 as control of the N. Cells were resuspended in 500 μl of solution containing 10 mM HEPES pH 7.9, 10 mM KCl, 1.5 mM $MgCl_2$, 0.1 mM EDTA, 0.1 mM DTT, 1× protease inhibitor and phosphatase inhibitor cocktail and incubated in ice for 10 min. Next, the solution was centrifuged and supernatant (extra nuclear fraction consisting of cytoplasm and membranes) was collected [40]. The nuclei fraction was prepared by adding a solution containing 50 mM Tris–HCl pH 8.0, 150 mM NaCl, 0.5% sodium deoxycholate, 0.1% SDS, 1 mM EDTA, 1× protease inhibitor and phosphatase inhibitor cocktail to pellet nuclei and then homogenized, using a syringe by ~10 iterations of up and down, and centrifuged (13000 rpm, 30 min). The-proteins were loaded on 10% SDS–PAGE and detected by western blotting. Supplementary material for Figure 4: (**B**) Specific gRNA sequence targeting the ATF6 mouse gene. (**C**) Western blotting showing the CRISPR/Cas9-induced knock-out efficiency both in the bulk culture (pool) and of the selected clones of C2C12 cell line.

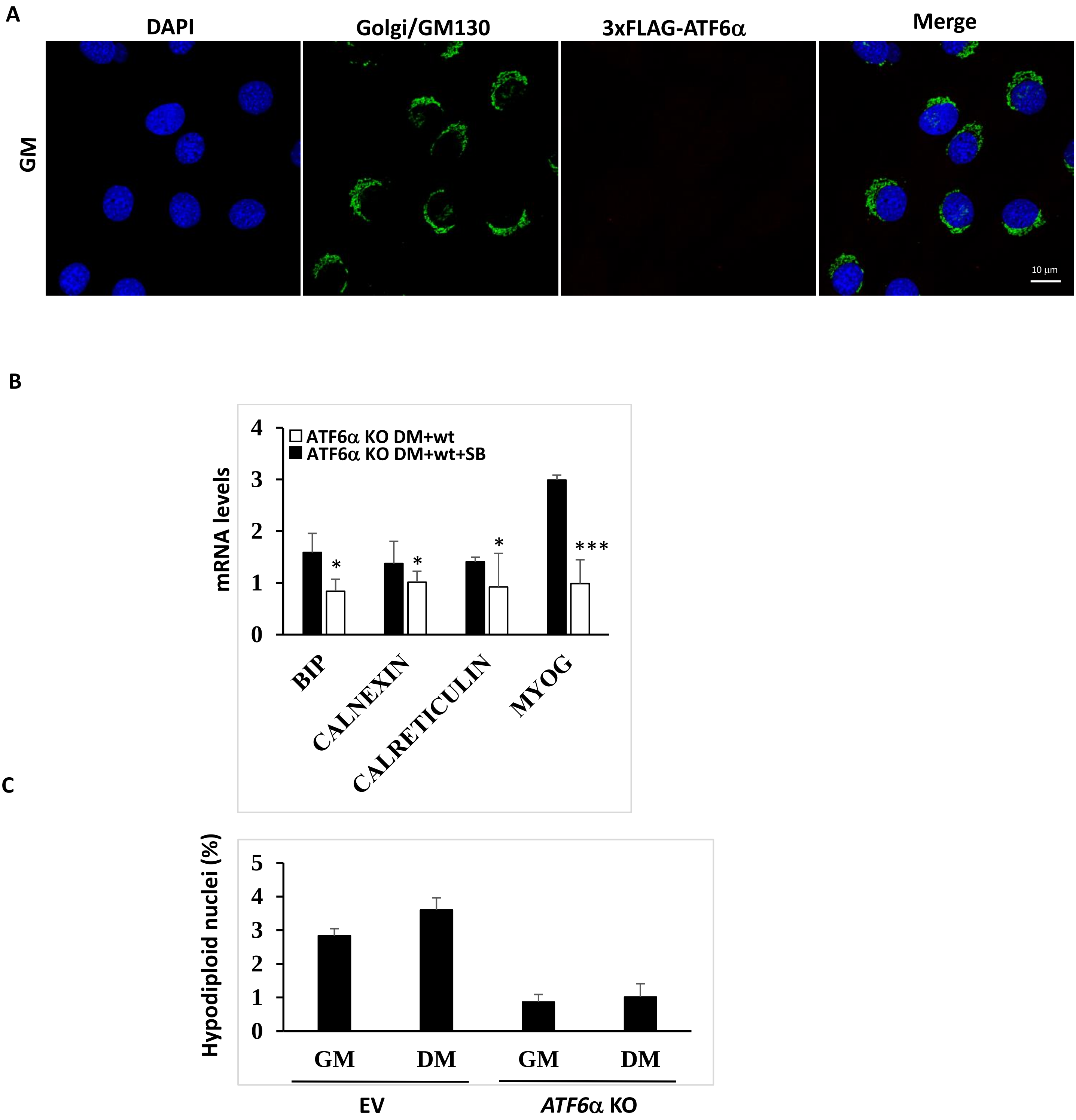

Figure S2. Supplementary material for Figures 5, 6 and 8. Supplementary material for Figure 5: (**A**) Un-transfected cells were used as a negative control for immunofluorescence experiment. Cells were subjected to fluorescence analysis with FLAG antibody (red) and GM130 to visualized the Golgi (green). Nuclei were stained with DAPI (blue). Scale bar = 10 µm. Supplementary Material for Figure 6: (**B**) Quantitative qPCR analysis of GRP78/BiP, Calnexin, Calreticulin and MyoG mRNA expression levels ATF6 KO cells treated or not with 10 µM of SB, inhibitor of p38, for 72 h of differentiation. 18S was used as the internal control. * and *** indicate values statistically different from 72 h + WT ($p \leq 0.05$; $p \leq 0.001$). Supplementary Material for Figure 8: (**C**) Cell lines were incubated in DM or kept in GM for 72 h. Apoptosis was evaluated after the indicated incubation time and reported as a percentage.

References

40. Gade, P.; Ramachandran, G.; Maachani, U.B.; Rizzo, M.A.; Okada, T.; Prywes, R.; Cross, A.S.; Mori, K.; Kalvakolanu, D.V. An IFN-gamma-stimulated ATF6-C/EBP-beta-signaling pathway critical for the expression of Death Associated Protein Kinase 1 and induction of autophagy. *Proc. Natl. Acad. Sci. USA* **2012**, *109*, 10316–10321. https://doi.org/10.1073/pnas.1119273109.
